# Neuroprotective Effects of a Variety of Pomegranate Juice Extracts against MPTP-Induced Cytotoxicity and Oxidative Stress in Human Primary Neurons

**DOI:** 10.1155/2013/685909

**Published:** 2013-10-03

**Authors:** Nady Braidy, Subash Selvaraju, Musthafa Mohamed Essa, Ragini Vaishnav, Samir Al-Adawi, Abdullah Al-Asmi, Hamed Al-Senawi, Ammar Abd Alrahman Alobaidy, Ritu Lakhtakia, Gilles J. Guillemin

**Affiliations:** ^1^Centre for Healthy Brain Ageing, School of Psychiatry, Faculty of Medicine, University of New South Wales, Sydney, NSW 2031, Australia; ^2^Department of Food Science and Nutrition, College of Agriculture and Marine Sciences, Sultan Qaboos University, P.O. Box 50, Muscat 123, Oman; ^3^Ageing and Dementia Research Group, Sultan Qaboos University, P.O. Box 50, Muscat 123, Oman; ^4^College of Medicine and Health Sciences, Sultan Qaboos University, P.O. Box 50, Muscat 123, Oman; ^5^Neuropharmacology Group, MND and Neurodegenerative Diseases Research Group, Australian School of Advanced Medicine (ASAM), Macquarie University, Sydney, NSW 2109, Australia

## Abstract

1-Methyl-4-phenyl-1,2,3,6-tetrahydropyridine (MPTP) is an environmental toxin which selectively induces oxidative damage and mitochondrial and proteasomal dysfunctions to dopaminergic neurons in the substantia nigra leading to Parkinsonian syndrome in animal models and humans. MPTP is one of the most widely used *in vitro* models to investigate the pathophysiology of Parkinson's disease (PD) and, screen for novel therapeutic compounds that can slow down or ameliorate this progressive degenerative disease. We investigated the therapeutic effect of pomegranate juice extracts (PJE), Helow, Malasi, Qusum, and Hamadh against MPTP-induced neurotoxicity in primary human neurons by examining extracellular LDH activity, intracellular NAD^+^ and ATP levels, and endogenous antioxidant levels including lipid peroxidation products, catalase, superoxide dismutase (SOD) and glutathione peroxidase (GPx) activities, and reduced glutathione (GSH) levels. MPTP induced a reduction in SOD and GPx activities and intracellular NAD^+^, ATP, and GSH levels parallel to an increase in extracellular LDH and CAT activities, although lipid peroxidation was not altered. We report that helow and malasi can ameliorate MPTP-induced neurotoxicity by attenuating the observed changes in redox function to a greater extent than qusum and hamedh. Selected PJE varieties may exhibit properties which may be of therapeutic value to slow down age-related degeneration and neurodegeneration in particular.

## 1. Introduction

As the ageing population continues to grow at an alarming rate, the likelihood of people developing debilitating neurodegenerative deficits such as Parkinson's disease (PD) is growing rapidly. PD represents the second most common neurological disorder after Alzheimer's disease (AD), and it affects 2% of the population over the age of 60. PD is characterised by the chronic and progressive loss of dopaminergic neurons in the substantia nigra [1]. Although the etiology of PD is not yet known, current studies have suggested that oxidative stress may be a major player [2]. An imbalance between the formation of free radicals and reactive oxygen species (ROS) and the body's endogenous antioxidant defense mechanisms has also been implicated in the pathogenesis of other neurodegenerative diseases such as AD, Huntington's disease (HD), Pick's disease, amyotrophic lateral sclerosis (ALS), epilepsy, schizophrenia, and hypoxic-ischemic brain injury. ROS can induce oxidative damage to lipids, nucleic acids, and proteins, promote abnormal aggregation of cytoskeletal proteins, inactivate major metabolic enzymes, and facilitate mitochondrial dysfunction and the formation of reactive nitrogen species (RNS) and advanced glycation end products formation leading to further oxidative stress formation [3–20]. Therefore, an increased total antioxidant capacity has been associated with protection against neurodegeneration [12].

The environmental toxin 1-methyl-4-phenyl-1,2,3,6-tetrahydropyridin (MPTP) can induce neurotoxicity to humans [21], subhuman primates, and mice [22–24] by eliciting damage to dopaminergic neurons and subsequently resulting in Parkinsonian-like syndrome in animals and humans. MPP^+^ (1-methyl-4-phenylpyridinium ion) represents the neurotoxic form of MPTP which is formed by 4-e oxidation of MPTP in brain mitochondria [25]. It remains unclear whether or not MPP^+^ is the main neurotoxic product of MPTP, and why other organs apart from the brain are not vulnerable to MPTP-induced cytotoxicity. The “mitochondrial hypothesis” suggests that MPP^+^ can inhibit mitochondrial respiration similar to the synthetic pesticide, rotenone. On the other hand, the “oxidative stress” hypothesis assumes that the nigrostriatal cell death observed in PD is due to the MPTP-mediated formation of hydroxyl and superoxide radicals. Nevertheless, the neuronal lesions and neurological symptoms induced by MPTP and its congeners are similar to those reported in idiopathic parkinsonism and provides additional evidence to suggest that environmental toxins of related structure may play a causal role in human PD [26, 27].

The pomegranate (*Punica granatum* L.) is a polyphenolic rich fruit that has been extensively referenced in medical folklore [28]. In several countries of the Arabian Peninsula and notably Yemen, pomegranates are widely used for the treatment of common ailments, including diarrhea, stomachache, healing wounds, acidosis, dysentery, microbial infections, haemorrhage, and various infectious and non-infectious respiratory pathologies [29]. Phytochemicals such as polyphenols (including the phenolic acids and flavonoids which are concentrated in pomegranates) have demonstrated antioxidant properties and can inhibit inflammation and other deleterious processes involved in degenerative diseases [30]. Pomegranate pericarp is also highly rich in tannins (gallic acid, ellagic acid), which are potent antioxidants [31]. These polyphenols have been shown to inhibit carcinogenesis [32] and display various anticancer properties [33]. Tannins which are present in high levels in commercially processed pomegranate juice from pressing the whole fruit and the peels also augment the juices antioxidant power [34].

The antinflammatory and antibacterial potentials of pomegranate have been previously reported [35–43]. Further research has demonstrated that polyphenols possess powerful antioxidant properties which represent the most likely mechanism responsible for pomegranate's protective benefits [41]. Although pomegranate juice extract has been previously shown to reduce amyloid load and improve cognitive behavioural deficits in mouse models for AD, little is known about the potential beneficial effects of pomegranates in PD. Therefore, we investigated whether various pomegranate extracts could protect against MPTP-induced oxidative stress in primary human neurons *in vitro*.

## 2. Materials and Methods

### 2.1. Pomegranate Juice Extracts (PJE) Preparation

Fresh pomegranate (Helow, Malasi, Qusum, and Hamedh) varieties were obtained from Al-Jabal Al-Akdhar farms, Oman. The seeds were isolated and ground to obtain juice for all varieties separately. The juices (PJ) were air-dried at 40°C and concentrated under reduced pressure to obtain PJ extract (PJE). Appropriate preparation of PJE is vital to prevent some undesirable reactions, such as enzymatic and non-enzymatic degradation, fat oxidation, vitamin degradation, and protein denaturation prior to experimentation. Drying is the most commonly used method for the dehydration of food products. Various fruits and vegetables such as onions, red pepper, garlic cloves, and apricots have been dried, although this led to a reduction in size and loss of colour, texture and nutritional-functional properties (reviewed in [44]). Our PJE were dried as previously described by Bchir et al. (2012) with the lowest impact on the gallic acid equivalent fresh matter (FM) of total phenolics, FM of anthocyanins, antioxidant activity, and texture [45] of the extract used in the study.

### 2.2. Measurement of Total Phenolics in Pomegranate Juice Extracts

Total phenolics of PJE were measured by the modified Folin-Ciocalteu assay as previously described [46]. Briefly, 250 *μ*L Folin-Ciocalteu reagent was mixed with 10 *μ*L of PJE. After a short incubation of 5 mins, 750 *μ*L of sodium carbonate (1.9 M) was added and incubated for 2 h at 25°C. The absorbance at 765 nm was measured and compared with that from gallic acid (GA) standards. The concentration of phenolics in pomegranate juice extracts was expressed as gallic acid equivalents (GAE). All the measurements were taken in triplicate, and the mean values were calculated.

### 2.3. Human Primary Neuronal Cell Cultures

Human foetal brains were obtained from 16- to 19 week-old foetuses collected following therapeutic termination with informed consent. Mixed brain cultures were prepared and maintained using a protocol previously described by Guillemin et al. [47]. Neurons were prepared from the same mixed brain cell cultures as previously described [48]. Briefly, cells were plated in 24-well culture plates coated with Matrigel (1/20 in Neurobasal) and maintained in Neurobasal medium supplemented with 1% B-27 supplement, 1% Glutamax, 1% antibiotic/antifungal, 0.5% HEPES buffer, and 0.5% glucose.

### 2.4. Cell Culture Treatments

Human neurons were divided into four groups: (1) control group: the cells were treated with 0.1% DMSO solution (control) alone; DMSO at the concentrations used had no effect on cell viability; (2) MPTP-treated group: the cells were treated with pathophysiological concentrations of MPTP (0.05 mM) for 24 hours; (3) PJE-treated group: the cells were treated with varieties of PJE at 1, 10, 50, and 100 *μ*M for 24 hours; (4) PJE/MPTP-treated group: neurons were pretreated with PJE at 1, 10, 50, and 100 *μ*M for 1 hour and then 0.05 mM MPTP (final concentration) was added for 24 hours into cultured neurons in 24-well plates containing supplemented Neurobasal medium as described above. Experiments were performed in quadruplicates using cultures derived from three different human foetal brains.

### 2.5. NAD(H) Microcycling Assay for the Measurement of Intracellular NAD^+^ Concentrations

Intracellular NAD^+^ concentration was measured spectrophotometrically using the thiazolyl blue microcycling assay established by Bernofsky and Swan [49] and adapted for 96-well plate format by Grant and Kapoor [50].

### 2.6. Extracellular LDH Activity as a Measurement for Cytotoxicity

The release of lactate dehydrogenase (LDH) into culture supernatant correlates with the amount of cell death and membrane damage, providing an accurate measure of cellular toxicity. LDH activity was assayed using a standard spectrophotometric technique described by Koh and Choi [51].

### 2.7. Measurement of Intracellular ATP Levels

Human neuronal cell lysates were collected by centrifugation, and intracellular ATP was measured with a luminometer using an ATP Bioluminescence Assay Kit HS II (Roche Molecular Biochemicals) according to the manufacturer's instructions. Briefly 50 *μ*L of reaction mixture was added to 50 *μ*L of cell homogenate and the count was measured by luminometer (BD Biosciences). The level of ATP was determined from the standard curve. The standard was prepared for each day of measurements.

### 2.8. Malondialdehyde-Thiobarbituric Acid (MDA) as a Marker for Lipid Peroxidation

The level of lipid peroxidation was quantified by measuring the amount of malondialdehyde-thiobarbituric acid (MDA-TBA) adduct formed by the reaction of MDA and TBA at 100°C in neuronal cell lysates. MDA levels were measured using a standardised commercial assay kit (Caymen Chemical Co. Ann Arbor, MI, USA) according to the manufacturer's instructions. Briefly, 50 *μ*L of sample was added to 50 *μ*L SDS solution and 1 mL TBA and incubated at 100°C for 1 hr. Afterwards, the samples were placed on ice for 10 minutes to terminate the reaction and centrifuged at 1600 g for 10 min to remove debris. The absorbance for the newly formed product was read at 540 nm using the Model 680XR microplate reader (BioRad, Hercules, USA).

### 2.9. Superoxide Dismutase Activity Assay

Superoxide dismutase (SOD) activity was assayed using a colorimetric assay kit (Cayman, MI, USA). The kit measures all three types of SOD (Cu/Zn-, Mn-, and Fe-SOD). The kit utilizes a tetrazolium salt for the detection of superoxide radicals generated by xanthine oxidase. One unit of SOD is defined as the amount of enzyme needed to exhibit 50% dismutation of the superoxide radical. The absorbance was read at 450 nm using the Model 680XR microplate reader (BioRad, Hercules).

### 2.10. Catalase Activity Assay

Catalase (CAT) activity was assayed by the decomposition of hydrogen peroxide using a colorimetric assay kit (Cayman, MI, USA). The method is based on a two-stage reaction. The rate of dismutation of H_2_O_2_ to water and molecular oxygen correlates with catalase activity. A known amount of H_2_O_2_ was added to the cell homogenate and incubated for exactly 1 minute. The reaction was stopped using sodium azide. The amount of H_2_O_2_ remaining in the reaction mixture was then determined by the oxidative coupling reaction of 4-aminophenazone (AAP) and 3,5-dichloro-2-hydroxybenzenesulfonic acid (DHBS) catalyzed by horseradish peroxidase. The resulting quinone imine dye was measured at 520 nm using the Model 680XR microplate reader (BioRad, Hercules).

### 2.11. Glutathione Peroxidase Activity Assay

The activity of glutathione peroxidase (GPx) was assayed using a colorimetric assay kit (Cayman, MI, USA). This assay is based on the principle that oxidized glutathione (GSSG) produced upon reduction of an organic peroxide by GPx is immediately converted to its reduced form (GSH) with concomitant oxidation of NADPH to NADP^+^. The oxidation of NADPH was monitored spectrophotometrically using the Model 680XR microplate reader (BioRad, Hercules) as a decrease in absorbance at 340 nm.

### 2.12. Estimation of Glutathione

 The total glutathione (GSH) content was measured using the reduced glutathione assay kit (Cayman, MI, USA). In this assay, o-phthalaldehyde (OPA) reacts with GSH present in the sample, and the fluorescence intensity (ex. 340 nm, em. 420 nm) was measured every 30 sec for a total of 60 min using Fluostar Optima Fluorometer (NY, USA).

### 2.13. Bradford Protein Assay for the Quantification of Total Protein

All assays were normalised for variations in cell number using the Bradford protein assay described by Bradford [52].

### 2.14. Data Analysis

Results obtained are presented as the means ± the standard error of measurement (SEM). One way analysis of variance (ANOVA) and post hoc Tukey's multiple comparison tests were used to determine statistical significance between treatment groups. Differences between treatment groups were considered significant if *P* was less than 0.05 (*P* < 0.05).

## 3. Results

### 3.1. Total Phenolic Content of Various Pomegranate Juice Extracts

Our analysis of polyphenolic derivatives in PJE indicates the presence of gallic acid equivalents (GAE) in the Helow, Malasi, Qusum and Hamedh varieties (Table 1). Helow and Malasi showed the highest GAE compared to Qusum and Hamadh, the latter showed the least GAE.

### 3.2. Neuroprotective Effects of Pomegranate Juice Extracts on MPTP-Induced Neurotoxicity

**Figure 1:**
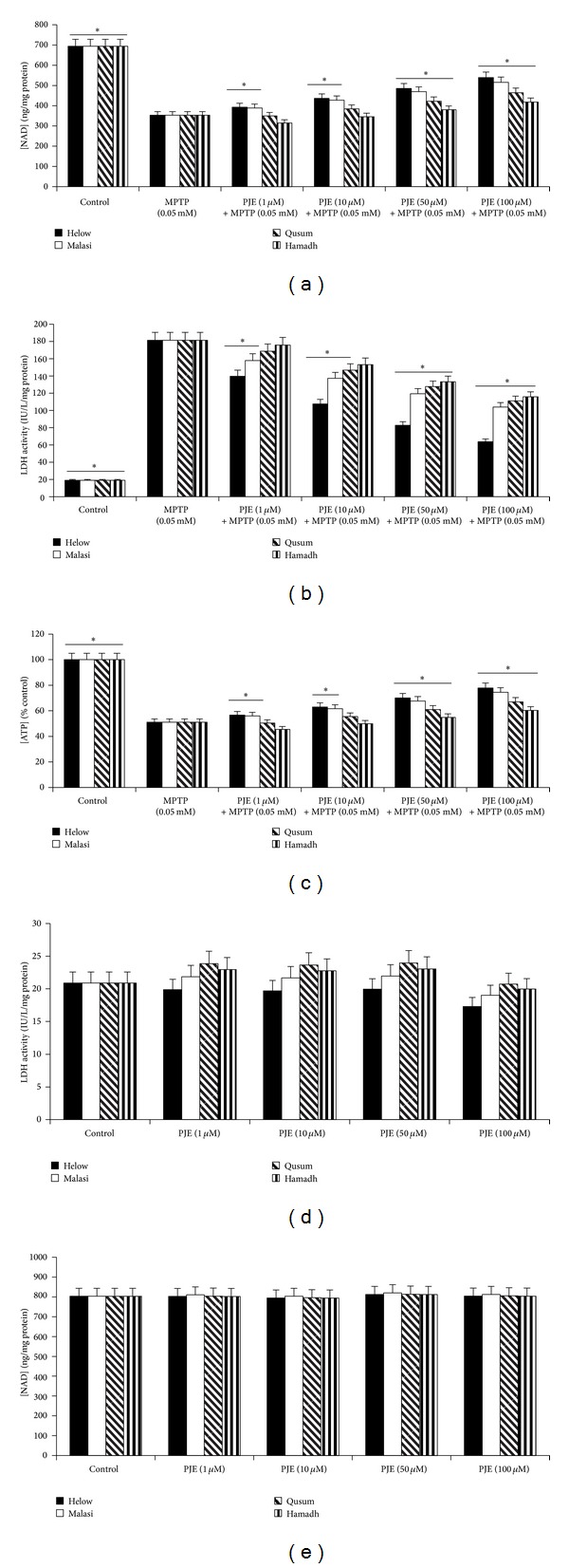
Effect of PJE on MPTP-induced NAD^+^ and ATP depletions and cell viability in human neurons. Effect of (a) Helow, Malasi, Qusum, and Hamadh varieties on NAD^+^ depletion in the presence of MPTP (0.05 mM) for 24 hours (*P < 0.05 compared with 0.05 mM MPTP alone); (b) Helow, Malasi, Qusum, and Hamadh varieties on extracellular LDH activity in the presence of MPTP (0.05 mM) (*P < 0.05 compared with 0.05 mM MPTP alone), *n* = 4 for each treatment group; (c) Helow, Malasi, Qusum, and Hamadh varieties on intracellular ATP levels in the presence of MPTP (0.05 mM) (*P < 0.05 compared with 0.05 mM MPTP alone), *n* = 4 for each treatment group; (d) Helow, Malasi, Qusum, and Hamadh varieties alone on extracellular LDH activity (*P < 0.05 compared with control), *n* = 4 for each treatment group; (e) Helow, Malasi, Qusum, and Hamadh varieties alone on intracellular NAD^+^ levels (*P < 0.05 compared with control), *n* = 4 for each treatment group.

Extracellular LDH activity and intracellular NAD^+^ levels were used as measurements of neurotoxicity in primary human foetal neurons. Our data shows that treatment with MPTP (0.05 mM) alone for 24 hours not only increased extracellular LDH activity, but also reduced intracellular NAD^+^ levels significantly. In contrast, pretreatment with all PJE varieties significantly reduced extracellular LDH activity (Figure 1(b)) and ameliorated the MPTP-mediated decline in intracellular NAD^+^ levels in a dose-dependent manner (Figure 1(a)). The Helow and Malasi varieties showed the greatest neuroprotective effect compared to Qusum and Hamadh. As well, MPTP treatment leads to a significant depletion of intracellular ATP levels after 24 hours (Figure 1(c)). Pretreatment with PJE increased ATP levels significantly, compared to MPTP treatment alone, with Helow and Malasi showing the greatest effect compared to that of Qusum and Hamadh. No significant changes in extracellular LDH activity (Figure 1(d)) and intracellular NAD^+^ levels (Figure 1(e)) were detected in neuronal cells treated with PJE alone. Thus, it was possible to conclude that PJE varieties were effective for the protection of human neurons against MPTP-mediated toxicity *in vitro*.

### 3.3. Effect of PJE Varieties on MPTP-Induced ROS Formation and Alterations to Antioxidant Enzyme Activity

**Figure 2:**
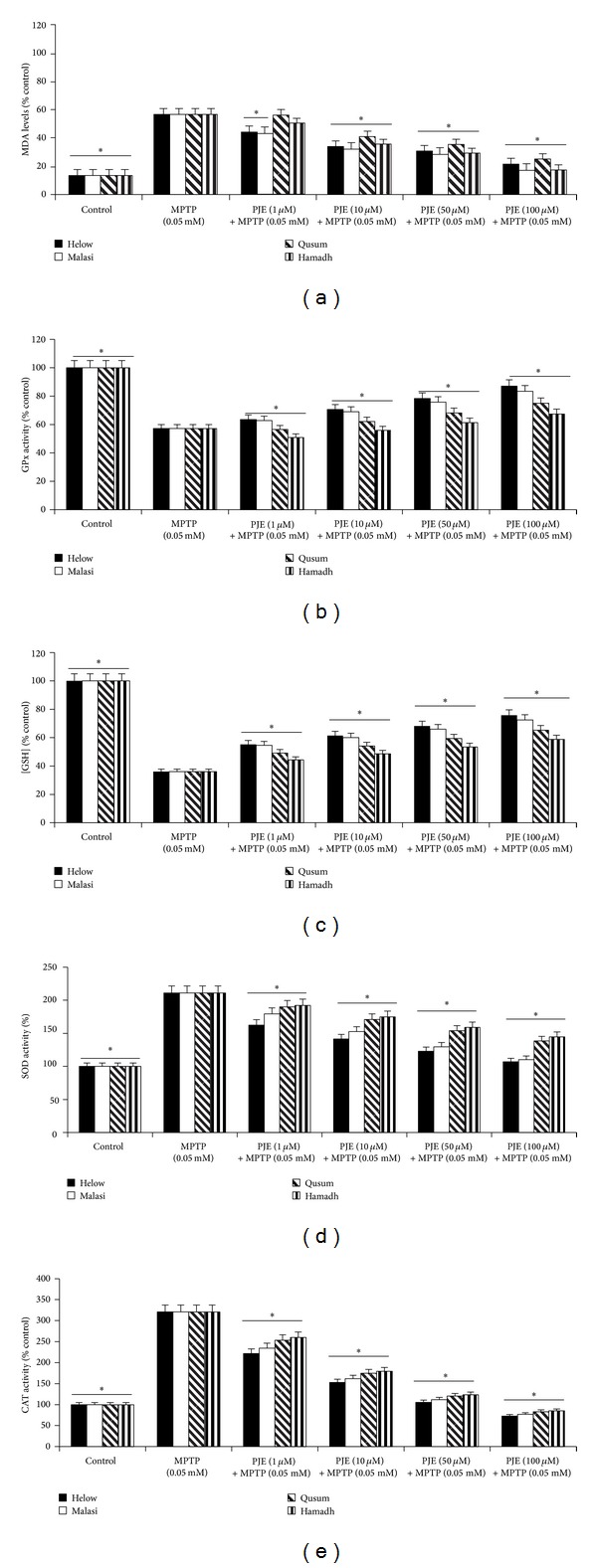
Effect of PJE on MPTP-induced increase in lipid peroxidation and MPTP-mediated changes to endogenous antioxidant activities in human neurons. Effect of (a) Helow, Malasi, Qusum, and Hamadh varieties on MDA levels in the presence of MPTP (0.05 mM) for 24 hours (*P < 0.05 compared with 0.05 mM MPTP alone); (b) Helow, Malasi, Qusum, and Hamadh varieties on GPx activity in the presence of MPTP (0.05 mM) for 24 hours (*P < 0.05 compared with 0.05 mM MPTP alone); (c) Helow, Malasi, Qusum, and Hamadh varieties on intracellular GSH levels in the presence of MPTP (0.05 mM) for 24 hours (*P < 0.05 compared with 0.05 mM MPTP alone); (d) Helow, Malasi, Qusum, and Hamadh varieties on SOD activity in the presence of MPTP (0.05 mM) for 24 hours (*P < 0.05 compared with 0.05 mM MPTP alone); (e) Helow, Malasi, Qusum, and Hamadh varieties on CAT activity in the presence of MPTP (0.05 mM) for 24 hours (*P < 0.05 compared with 0.05 mM MPTP alone).

Since free radicals are thought to play a major role in the mechanism(s) of MPTP-induced neurotoxicity, we investigated whether MPTP administration can increase formation of lipid peroxidation and alter the activity of the endogenous antioxidant enzymes, superoxide dismutase (SOD), catalase (CAT) and glutathione peroxidase (GPx), and glutathione (GSH) levels *in vitro*. Interestingly, MPTP treatment alone (0.05 mM) did not significantly increase the levels of malondialdehyde (MDA), and no significant effect in MDA levels was observed in PJE-pretreated cells (Figure 2(a)). However, a significant reduction in GPx (Figure 2(b)) activities and GSH levels (Figure 2(c)) was observed following MPTP treatment, which was attenuated when the cells were pretreated with PJE in a dose-dependent manner. In contrast, total SOD (Figure 2(d)) and CAT activities (Figure 2(e)) increased by one- and two-fold, respectively, in neuronal cells treated with MPTP, and the effect was reduced following pretreatment of MPTP-treated cells with PJE in a dose-dependent manner. Again, Helow and Malasi showed the greatest antioxidant effect compared to Qusum and Hamadh.

## 4. Discussion

Although several new therapies have emerged to treat PD, these treatment strategies only provide symptomatic relief and do not affect the progression of the disease [53]. Moreover, long-term use of these drugs can induce adverse side effects which may not be tolerated by patients with PD. Therefore, newer, more effective drugs that specifically target PD development are needed. Recent studies have focused on the benefits of naturally occurring phytochemicals that exhibit potent antioxidant effects as potential neuroprotective agents [54, 55]. Pomegranate has antioxidant function that may help protect neurons against MPTP-induced neurotoxicity. The neuroprotective effects of PJE have been previously demonstrated using an *in vivo* transgenic animal model of AD [56]. However, the effects of PJE on MPTP-induced neurotoxicity in primary human neuronal cells have not been investigated previously.

The biotransformation of MPTP into MPP^+^, which is catalyzed by the mitochondrial enzyme monoamine oxidase B, represents the major route for MPTP-mediated neurotoxicity [57]. The conversion of MPTP to MPP^+^ has been suggested to induce the formation of ROS. This notion is supported by previous studies which showed increased superoxide (O_2_^•−^) and hydroxyl radical (^•^OH) levels during the biotransformation of MPTP (reviewed in [58]). While the damage induced by O_2_^•−^ is limited, it can react with nitric oxide (NO) to form peroxynitrite (ONOOO^−^) which readily forms the more reactive ^•^OH radical. Other studies have shown that MPTP induces toxicity through ATP depletion and mitochondrial dysfunction. Moreover, Kutty et al. (1991) showed that ATP depletion plays a major role in MPTP-induced neuronal cell death [59].

In this paper, we showed that selected PJE can protect against MPTP-induced neurotoxicity in primary human neurons in a dose-dependent manner by attenuating MPTP-induced increase in extracellular LDH activity. However, we did not observe a significant increase in lipid peroxidation, an established measure for oxidative stress. MDA is widely used to assess lipid peroxidation both *in vitro* and *in vivo* [60]. However, it is likely that MDA can form complexes with other biological components such as protein, lipids, and nucleic acids which can contribute to an underestimation of endogenous lipid peroxidation [61]. On the contrary to our lipid peroxidation data, we also show that MPTP can lead to distinct alterations in endogenous antioxidant defense mechanisms. MPTP treatment has been previously shown to significantly increase Mn-SOD and CuZn-SOD activities in the striatum of C57BL/6 mice, which is suggestive of acute oxidative stress insult [62]. SOD is upregulated in cells when O_2_^•−^ is produced in excessive levels [63]. This observation suggests that SOD may play a role in the toxicity observed following acute treatment of MPTP, although ROS formation may not play a major role in MPTP-induced toxicity. We also observed a significant increase in CAT after a 24-hour treatment with MPTP. CAT is an enzyme that is involved in the detoxification of ROS and the elimination of hydrogen peroxide (H_2_O_2_) in particular [64]. The increase in both intracellular SOD and CAT activities may therefore represent an adaptive response due to the leakage of free radicals during impaired mitochondrial respiration.

 Treatment with MPTP also leads to reduced activity of GPx and decreased levels of the essential pyridine nucleotide NAD^+^, ATP, and GSH in primary human neurons after a 24-hour exposure. The maintenance of GPx activity appears crucial for the maintenance of cell viability during oxidative insult [65–68]. One study showed that increased GPx expression could protect against H_2_O_2_-mediated oxidative stress due to methamphetamine as measured by extracellular LDH activity [69]. Moreover, previous studies have shown that MPP^+^, the metabolite of MPTP induces GSH depletion without increasing the levels of oxidized glutathione disulfide (GSSG) [70]. Reduced GSH levels may occur due to that MPP^+^ induced decline in intracellular NAD^+^ and ATP stores which are necessary for GSH anabolism, release, and consequent hydrolysis. Taken together, our data suggests that MPTP exposure can limit the endogenous antioxidant defense, subsequently increasing the vulnerability of neuronal cells to additional oxidative stress. An imbalance in the function of endogenous antioxidant defense mechanisms can lead to the accumulation of free radicals and ROS and increased susceptibility to oxidative stress, which contributes to the pathogenesis of PD.

Different brain cell types are used to study the effects of oxidative stress in culture. Although our data relate to fetal neuronal cultures, it is likely that our results also reflect what is happening in adult astrocytes and neurons. Human cell culture models have demonstrated a neuroprotective role of both astrocytes and microglial cells against ROS mediated neuronal cell death. However, at the same time, evidence exist, linking neurotoxicity to oxidative stress mediated astrocyte/microglial activation [71, 72]. We have previously shown that the inflammatory profile is conserved between human and simian adult and foetal astrocytes and neurons [73, 74]. Therefore, human foetal brain cells are a relevant model to study neurodegenerative diseases and MPTP-induced toxicity in particular.

PJE are known to exhibit antioxidant and anti-inflammatory properties. PJE have been shown to have a variety of protective effects in several disease models, including reduced low-density lipoprotein (LDL) aggregation, oxidative stress, amyloid load, and improved cognitive behaviour in AD mice [35–43]. Nevertheless, the effect of PJE against the MPTP toxicity in human neurons has not been previously investigated. Our data shows that PJE can reverse the effect of MPTP on the activities of antioxidant enzymes and attenuate MPTP-induced toxicity in a dose-dependent manner. Helow and Malasi varieties showed a more potent effect against MPTP toxicity compared to Qusum and Hamadh. An assessment of total phenolic compounds present in these varieties suggests that the latter have the lowest concentration of phenolics compared to Helow and Malasi varieties and can explain the lower protective effects observed by Qusum and Hamadh. The neuroprotective effects of polyphenols have been associated with their antioxidant and free-radical scavenging, iron/metal chelating ability, as well as their anti-inflammatory properties [75–78].

Our findings show that PJE at the stated concentrations have no toxic effect on human neurons and may therefore be therapeutically safe. However, little information is available in the literature regarding the absorption, bioavailability, biodistribution, and metabolism of important bioactive constituents found in PJE, such as phenolic acids, flavonoids, and tannins [79]. An *in vitro* study of the digestion of pomegranate phenolic compounds showed that these molecules are present during digestion in relatively large amounts (29%). However, anthocyanins are metabolised or degraded (97%). Seeram et al. (2008) investigated the bioavailability of polyphenols derived from PJE in liquid and lypophilised form. Plasma bioavailability, determined by examining GAE levels 6 hours after consumption, was not statistically different between the 2 interventions. The time of maximum concentration was delayed in the polyphenol powder extract (2.58 ± 0.42 h) compared with that of pomegranate juice (0.65 ± 0.23 h) and polyphenol liquid extract (0.94 ± 0.06 h) [80]. It is likely that the bioavailability of pomegranate polyphenols may be affected by several factors, including individual variability, differential processing of pomegranate juice, and the analytical techniques employed to detect low postprandial concentrations of these metabolites [81].

 In conclusion, PJE provide protection against the neurotoxic effects of MPTP in human neurons, and the mechanisms of protection may be related to their antioxidant activity and botanical phenolic constituents. The potential neuroprotective effects of PJE warrant further investigation.

## Figures and Tables

**Table 1:** Gallic acid equivalent content in selected varieties of pomegranate juice extracts from Oman.

Name of pomegranate varieties	GAE (mg/100 g)
Helow	572.739 ± 0.261
Malasi	544.155 ± 0.506
Qusum	314.452 ± 0.086
Hamadh	281.671 ± 0.101

GAE: gallic acid equivalent.
